# Induction chemotherapy followed by chemoradiotherapy *versus* chemoradiotherapy alone as neoadjuvant treatment for locally recurrent rectal cancer: study protocol of a multicentre, open-label, parallel-arms, randomized controlled study (PelvEx II)

**DOI:** 10.1093/bjsopen/zrab029

**Published:** 2021-06-05

**Authors:** PelvEx Collaborative, E L K Voogt, S Nordkamp, A G J Aalbers, T Buffart, G J Creemers, C A M Marijnen, C Verhoef, K Havenga, F A Holman, M Kusters, A W K S Marinelli, J Melenhorst, N Abdul Aziz, N Abecasis, M Abraham-Nordling, T Akiyoshi, W Alberda, M Albert, M Andric, E Angenete, A Antoniou, R Auer, K K Austin, O Aziz, R P Baker, M Bali, G Baseckas, B Bebington, M Bedford, B K Bednarski, G L Beets, R G H Beets-Tan, M Berbée, J Berg, P L Berg, J Beynon, S Biondo, J G Bloemen, K Boyle, L Bordeianou, A B Bremers, M Brunner, P Buchwald, A Bui, A Burgess, D Burling, E Burns, N Campain, S Carvalhal, L Castro, A Caycedo-Marulanda, H M Ceha, K K L Chan, G J Chang, M Chang, M H Chew, A K Chok, P Chong, H K Christensen, H Clouston, M Codd, D Collins, A J Colquhoun, A Corr, M Coscia, M Cosimelli, P E Coyne, A S L P Crobach, R M P H Crolla, R S Croner, L Damjanovic, I R Daniels, M Davies, R J Davies, C P Delaney, M A J de Roos, J H W de Wilt, M D den Hartogh, Q Denost, P Deseyne, C Deutsch, R de Vos tot Nederveen Cappel, M de Vries, M Dieters, D Dietz, S Domingo, M Doukas, E J Dozois, M Duff, T Eglinton, J M Enrique-Navascues, E Espin-Basany, M D Evans, B Eyjólfsdóttir, M Fahy, N S Fearnhead, S Feshtali, K Flatmark, F Fleming, J Folkesson, F A Frizelle, J E Frödin, M A Gallego, E Garcia-Granero, J L Garcia-Sabrido, K Geboes, L Gentilini, M L George, V George, L Ghouti, F Giner, N Ginther, T Glyn, R Glynn, T Golda, H I Grabsch, B Griffiths, D A Harris, J AW Hagemans, V Hanchanale, D P Harji, R M Helewa, H Helgason, G Hellawell, A G Heriot, S Heyman, D Hochman, C Hoff, W Hohenberger, T Holm, R Hompes, K Horsthuis, G Hospers, J Houwers, H Iversen, J T Jenkins, S Kaffenberger, G V Kandaswamy, S Kapur, Y Kanemitsu, G Kats-Ugurlu, S R Kelley, D S Keller, M E Kelly, K Keymeulen, M S Khan, H Kim, H J Kim, C E Koh, N F M Kok, R Kokelaar, C Kontovounisios, H Ø Kristensen, H M Kroon, S Kumar, V Lago, Z Lakkis, T Lamberg, S G Larsen, D W Larson, W L Law, S Laurberg, P J Lee, M M Leseman-Hoogenboom, M Limbert, M L Lydrup, A Lyons, A C Lynch, C Mantyh, K L Mathis, C F S Margues, A Martling, O W M Meijer, W J H J Meijerink, A Merchea, S Merkel, A M Mehta, D R McArthur, F D McDermott, J S McGrath, S Malde, A Mirnezami, J RT Monson, J R Morton, J Nederend, I Negoi, J W M Neto, J L Ng, B Nguyen, M B Nielsen, G A P Nieuwenhuijzen, P J Nilsson, M L Nilsson, S Oei, A Oliver, S T O’Dwyer, V Oppedijk, G Palmer, E Pappou, J Park, D Patsouras, G Pellino, A C Peterson, H M U Peulen, G Poggioli, D Proud, M Quinn, A Quyn, N Rajendran, R W Radwan, S Rasheed, P C Rasmussen, E Rausa, S E Regenbogen, A Renehan, M C Richir, R Rocha, M Rochester, J Rohila, J Rothbarth, M Rottoli, C Roxburgh, T Rozema, B Safar, P M Sagar, A Sahai, A Saklani, T Sammour, R Sayyed, A M P Schizas, E Schwarzkopf, V Scripcariu, C Selvasekar, I Shaikh, D Shida, A Simpson, T Skeie-Jensen, J J G Slangen, N J Smart, P Smart, J J Smith, P Snaebjornsson, A M Solbakken, M J Solomon, M M Sørensen, L Sorrentino, F M Speetjens, E J Spillenaar Bilgen, S R Steele, D Steffens, K Stitzenberg, L Stocchi, N A Stylianides, T Swartling, H Sumrien, P A Sutton, T Swartking, E J Tan, C Taylor, P P Tekkis, J Teras, V Terpstra, R Thurairaja, E L Toh, P Tsarkov, Y Tsukada, S Tsukamoto, J J Tuech, W H Turner, J B Tuynman, E B van Duyn, W M U van Grevenstein, N C T van Grieken, L van Iersel, G van Lijnschoten, E van Meerten, G H van Ramshorst, H L van Westreenen, D van Zoggel, W Vasquez-Jimenez, L A Velema, E Verdaasdonk, H M W Verheul, K S Versteeg, G Vizzielli, K Uehara, C Wakeman, S Warrier, H H Wasmuth, K Weber, M R Weiser, J M D Wheeler, N A T Wijffels, J Wild, J M W E Willems, M Wilson, D C Winter, A Wolthuis, M L Wumkes, H Yano, B Yip, J Yip, R N Yoo, M A Zappa, D D E Zimmerman, H J T Rutten, J W A Burger

## Abstract

**Background:**

A resection with clear margins (R0 resection) is the most important prognostic factor in patients with locally recurrent rectal cancer (LRRC). However, this is achieved in only 60 per cent of patients. The aim of this study is to investigate whether the addition of induction chemotherapy to neoadjuvant chemo(re)irradiation improves the R0 resection rate in LRRC.

**Methods:**

This multicentre, international, open-label, phase III, parallel-arms study will enrol 364 patients with resectable LRRC after previous partial or total mesorectal resection without synchronous distant metastases or recent chemo- and/or radiotherapy treatment. Patients will be randomized to receive either induction chemotherapy (three 3-week cycles of CAPOX (capecitabine, oxaliplatin), four 2-week cycles of FOLFOX (5-fluorouracil, leucovorin, oxaliplatin) or FOLFORI (5-fluorouracil, leucovorin, irinotecan)) followed by neoadjuvant chemoradiotherapy and surgery (experimental arm) or neoadjuvant chemoradiotherapy and surgery alone (control arm). Tumours will be restaged using MRI and, in the experimental arm, a further cycle of CAPOX or two cycles of FOLFOX/FOLFIRI will be administered before chemoradiotherapy in case of stable or responsive disease. The radiotherapy dose will be 25 × 2.0 Gy or 28 × 1.8 Gy in radiotherapy-naive patients, and 15 × 2.0 Gy in previously irradiated patients. The concomitant chemotherapy agent will be capecitabine administered twice daily at a dose of 825 mg/m^2^ on radiotherapy days. The primary endpoint of the study is the R0 resection rate. Secondary endpoints are long-term oncological outcomes, radiological and pathological response, toxicity, postoperative complications, costs, and quality of life.

**Discussion:**

This trial protocol describes the PelvEx II study. PelvEx II, designed as a multicentre, open-label, phase III, parallel-arms study, is the first randomized study to compare induction chemotherapy followed by neoadjuvant chemo(re)irradiation and surgery with neoadjuvant chemo(re)irradiation and surgery alone in patients with locally recurrent rectal cancer, with the aim of improving the number of R0 resections.

## Introduction

Locally recurrent rectal cancer (LRRC) occurs in 6–10 per cent of patients who undergo intentionally curative surgery for primary rectal cancer^1^^,^^2^. To cure patients with LRRC, achieving a resection with clear resection margins (R0 resection) is imperative^2–8^. When an R0 resection is achieved, 5-year overall survival rates vary between 48 and 58 per cent, whereas a resection without clear resection margins (R1/2 resection) results in a 5-year survival rate of only 10–18 per cent. Moreover, incomplete resections are associated with 5-year local re-recurrence rates of 70–80 per cent, and often result in severe morbidity, poor quality of life, and/or death^5^^,^^6^^,^^9–13^. Unfortunately, the attempt to achieve an R0 resection often fails because of challenging anatomy due to previous surgery, the presence of fibrosis as a result of previous radiotherapy, and the involvement of other structures such as adjacent organs, pelvic sidewall, and sacrum. To increase the chance of achieving an R0 resection, neoadjuvant treatment with chemoradiotherapy is considered the standard of care in many institutions^14^. In patients who received pelvic radiotherapy previously, reirradiation with a dose of 30 Gy has been proven to be safe and effective^3^^,^^15^. Despite the use of neoadjuvant chemo(re)irradiation, R0 resections are achieved in only 60 per cent of patients^16^^,^^17^. Therefore, there is ongoing research to optimize the treatment strategy for patients with LRRC.

### Potential benefits and disadvantages of induction chemotherapy

Induction chemotherapy in addition to neoadjuvant chemo(re)irradiation has the potential to induce more local tumour downstaging than can be achieved with chemoradiotherapy alone owing to the supplementary effect of the induction chemotherapy, and possibly also the synergistic effect of induction chemotherapy and chemoradiotherapy^18^. Improved local downstaging may subsequently increase the R0 resection rate, which has been identified as the main prognostic factor for overall survival^6–8^. When local downstaging is excellent, a pathologic complete response (pCR) can be achieved, which is a predictive variable for survival in patients with LRRC^19^. With improved local downstaging, the proportion of patients with a pCR may also increase. Alongside the local effect, induction chemotherapy may also have the potential to eradicate micrometastases^20^.

The addition of induction chemotherapy also has potential drawbacks. First, induction chemotherapy is associated with toxicity^21^. Second, chemotherapy-induced morbidity could delay, reduce or prevent subsequent treatment with chemoradiotherapy and surgery. Third, when chemoradiotherapy is preceded by induction chemotherapy, the toxicity of chemoradiotherapy may be increased. Finally, the prolonged and intensified neoadjuvant course may influence the patient’s performance status and may have a negative effect on surgical morbidity and mortality rates. Furthermore, prolonged neoadjuvant treatment may increase the risk of disease progression and secondary unresectability.

### Current evidence

Induction chemotherapy, whether or not combined with neoadjuvant chemoradiotherapy, is increasingly being used in the treatment of LRRC, although evidence for this approach is lacking^22^. Several retrospective studies and phase II clinical trials^23–26^ performed to investigate the role of induction chemotherapy in patients with primary locally advanced rectal cancer (LARC) have reported high R0 resection rates. However, other studies^27–29^, including comparative studies, did not demonstrate superior R0 resection rates after the addition of induction chemotherapy to neoadjuvant treatment.

Several studies investigating this treatment regimen in LARC used pCR as the primary endpoint. As in the studies focusing on R0 resection rate, the results were mixed. Some studies described promising pCR rates, whereas others found no effect of adding induction chemotherapy with regard to the pCR rate^30–34^. Regardless of the effect of this treatment on the R0 resection or pCR rate, induction chemotherapy seemed feasible, with high rates of compliance with the chemotherapy as well as with the subsequent chemoradiotherapy, and acceptable toxicity and postoperative morbidity^28^^,^^33^^,^^35^.

The available literature regarding induction chemotherapy in addition to chemoradiotherapy for patients with LRRC is limited; currently only three retrospective studies^19^^,^^36^^,^^37^ have been published. The first study^36^, which focused on patients with lateral local recurrence, reported a high R0 resection rate of 85 per cent in a subgroup of 13 patients who were treated with induction chemotherapy followed by chemoradiotherapy. In the second and third studies^19^^,^^37^, 58 and 132 patients respectively underwent induction chemotherapy followed by chemo(re)irradiation. Both studies reported promising pCR rates of 17 per cent, but the R0 resection rates did not appear to have improved. However, in both studies induction chemotherapy was initially administered to patients with unresectable disease or prognostically unfavourable characteristics, which may have had a negative impact on the R0 resection rate.

### Rationale for the study

Although the real benefit provided by the addition of induction chemotherapy to chemoradiotherapy and surgery for LRRC has not yet been established, its use is nevertheless increasing^22^.

This study will randomize patients with LRRC to receive either induction chemotherapy followed by chemoradiotherapy and surgery (experimental arm) or chemoradiotherapy and surgery alone (control arm). As R0 resection is the single most important prognostic factor for survival in patients with LRRC, the main hypothesis to be tested will be an increase in the R0 resection rate in the experimental arm compared with the control arm.

## Methods

### Study design and setting

This is a multicentre, international, open-label, phase III, parallel-arms study that will randomize eligible patients in a 1 : 1 ratio to receive either induction chemotherapy followed by neoadjuvant chemoradiotherapy and surgery (experimental arm) or neoadjuvant chemoradiotherapy and surgery alone (control arm). The study is registered with ClinicalTrials.gov (NCT04389086), including the list of centres enrolling for the trial. Surgical treatments will be limited to centres that perform at least 10 resections of LRRC per year (expert centres). Induction chemotherapy and chemoradiotherapy will be administered in expert centres and selected non-expert centres. This is protocol version 4.0, dated 10 December 2020.

### Participants

Patients aged 18 years or older, with resectable histopathologically or clinically proven LRRC after previous partial or total mesorectal resection, with a WHO performance status of 1 or less will be eligible for study participation. Patients with distant metastases at the time of randomization or in the previous 6 months, those who have undergone chemotherapy and/or radiotherapy in the past 6 months, patients with any contraindication to chemotherapy and/or radiotherapy and/or surgery, and those with concurrent malignancies that interfere with the planned study treatment or the prognosis of resected LRRC, will be excluded.

### Recruitment

Participants will be identified either by physicians in expert centres, or by physicians in non-expert centres who then refer the patients to an expert centre. All eligible patients will be reviewed in a multidisciplinary team (MDT) meeting in an expert centre to assess whether the patient meets the inclusion and exclusion criteria. The multicentre, international involvement in this study will ensure adequate participant enrolment to reach the targeted sample size.

### Interventions

Eligible patients who have signed informed consent will be randomized by the coordinating investigator in a 1 : 1 ratio using a software randomization program (ALEA Clinical; FormsVision, Abcoude, the Netherlands). Patients will be stratified for previous chemotherapy, previous radiotherapy, and expert centre. After randomization, the treating surgical oncologist will refer the patient to the medical oncologist (experimental arm) or radiation oncologist (control arm).

**Fig. 1:**
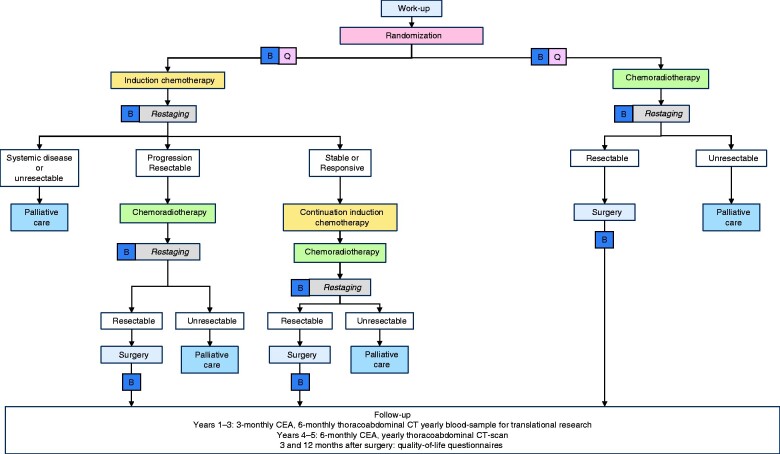
Study flow chart B, blood samples for translational research; Q, questionnaires; CEA, carcinoembryonic antigen.

The study flow chart is shown in *Fig. 1*, and study interventions and timelines for patients allocated to the experimental and control arms in *Tables 1* and *2* respectively.

**Table 1:** Schedule interventions and assessments experimental arm

	Before allocation	After allocation			Follow-up	
	Outpatient clinic	Induction chemotherapy	Chemoradiotherapy	Surgery	Years 1–3	Years 4–5
**Screening**						
Eligibility screen	☒					
Informed consent	☒					
Randomization	☒					
**Interventions**						
Induction chemotherapy		☒				
Chemoradiotherapy			☒			
Surgery				☒		
Thoracoabdominal CT	☒	☒^*^	☒^†^		☒^¶^	☒^††^
Pelvic MRI	☒	☒^*^	☒^†^			
Questionnaires	☒				☒^#^	
CEA level					☒^**^	☒^¶^
Translational research: blood	☒	☒^*^	☒^‡^	☒^§^	☒^††^	
Translational research: tissue				☒		
**Assessments**						
Baseline characteristics	☒					
Toxicity of induction chemotherapy		☒				
Toxicity of chemoradiotherapy			☒			
Radiological response		☒	☒			
Pathological response				☒		
Surgical characteristics				☒		
Postoperative morbidity				☒		
Progression-free survival					☒^**^	☒^¶^
Local recurrence-free survival					☒^**^	☒^¶^
Disease-free survival					☒^**^	☒^¶^
Overall survival					☒^**^	☒^¶^
Quality of life	☒				☒^#^	
Costs	☒				☒^#^	

*After three (CAPOX) or four (FOLFOX/FOLFIRI) cycles;

† 4–6 weeks after finishing chemoradiotherapy;

‡ after finishing chemoradiotherapy and before surgery;

§ 3 months after surgery;

¶ 6-monthly;

# 3 and 12 months after surgery;

** 3-monthly;

†† yearly. CEA, carcinoembryonic antigen.

**Table 2:** Schedule interventions and assessments control arm

	Before allocation	After allocation		Follow-up	
	Outpatient clinic	Chemoradiotherapy	Surgery	Year 1–3	Year 4–5
**Screening**					
Eligibility screen	☒				
Informed consent	☒				
Randomization	☒				
**Interventions**					
Induction chemotherapy					
Chemoradiotherapy		☒			
Surgery			☒		
Thoracoabdominal CT	☒	☒^*^		☒^§^	☒^**^
Pelvic MRI	☒	☒^*^			
Questionnaires	☒			☒^¶^	
CEA level				☒^#^	☒^§^
Translational research: blood	☒	☒^†^	☒^‡^	☒^**^	
Translational research: tissue			☒		s
**Assessments**					
Baseline characteristics	☒				
Toxicity of chemoradiotherapy		☒			
Radiological response		☒			
Pathological response			☒		
Surgical characteristics			☒		
Postoperative morbidity			☒		
Progression-free survival				☒^#^	☒^§^
Local recurrence-free survival				☒^#^	☒^§^
Disease-free survival				☒^#^	☒^§^
Overall survival				☒^#^	☒^§^
Quality of life	☒			☒^¶^	
Costs	☒			☒^¶^	

* 4 to 6 weeks after finishing chemoradiotherapy;

† after finishing chemoradiotherapy and before surgery;

‡ 3 months after surgery;

§ 6-monthly;

¶ 3 and 12 months after surgery;

# 3-monthly;

** yearly. CEA, carcinoembryonic antigen.

#### Induction chemotherapy

Patients allocated to the experimental arm will start treatment with induction chemotherapy within 4 weeks after randomization. Induction chemotherapy will consist of either three 3-week cycles of CAPOX (oxaliplatin 130 mg per m^2^ body-surface area (BSA) intravenously (i.v.) on day 1, capecitabine 1000 mg per m^2^ BSA, orally, twice daily on days 1–14), four 2-week cycles of FOLFOX (85 mg per m^2^ BSA of oxaliplatin i.v. on day 1, 400 mg per m^2^ BSA of leucovorin i.v. on day 1, 400 mg per m^2^ BSA of bolus 5-fluorouracil i.v. on day 1 followed by 2400 mg per m^2^ BSA of continuous 5-fluorouracil i.v. on days 1–2), or four 2-week cycles of FOLFIRI (180 mg per m^2^ BSA of irinotecan i.v. on day 1, 400 mg per m^2^ BSA of leucovorin i.v. on day 1, 400 mg per m^2^ BSA of bolus 5-fluorouracil i.v. on day 1 followed by 2400 mg per m^2^ BSA of continuous 5-fluorouracil i.v. on days 1–2). The choice of chemotherapy agent will be left to the physician’s discretion.

After three cycles of CAPOX or four cycles of FOLFOX or FOLFIRI, pelvic MRI will be performed for local restaging, and high-dose thoracoabdominal CT for restaging of possible distant metastases. Restaging imaging will be discussed during a dedicated MDT meeting in one of the expert centres. If a patient develops distant metastases or local disease becomes unresectable, best palliative treatment will be offered according to the standard of care. If a patient has progressive local disease, but surgery is still considered feasible, no further systemic therapy will be administered and patients will start treatment with chemoradiotherapy. If a patient has stable or responsive disease, induction chemotherapy will be continued with either one 3-week cycle of CAPOX or two 2-week cycles of FOLFOX or FOLFIRI.

#### Chemoradiotherapy

Patients in the experimental arm will start chemoradiotherapy within 3–5 weeks after the first day of the last cycle of chemotherapy. Patients in the control arm will start chemoradiotherapy within 4 weeks after randomization. The radiotherapy dose will depend on whether the patient received radiotherapy previously. In radiotherapy-naive patients, full-course radiotherapy will consist of 25 × 2.0 Gy or 28 × 1.8 Gy radiotherapy. In patients with a history of radiotherapy, the radiotherapy dose will consist of 15 × 2.0 Gy. The target volume will be defined by the gross, clinical, and planning target volumes (GTV, CTV and PTV respectively), and will be similar for radiotherapy-naive and previously irradiated patients. The GTV contains all macroscopic visible tumour, the CTV includes the GTV with a margin of 1 cm, without adjustment of the CTV towards other organs, and the PTV includes the CTV with a margin that can be determined according to local policy. Concomitant chemotherapy will comprise capecitabine, administered orally at a dose of 825 mg/m^2^ twice daily on radiotherapy days. In the event of unacceptable toxicity caused by capecitabine during induction chemotherapy, concomitant tegafur/gimeracil/oteracil administered orally at a dose of 25 mg/m^2^ twice daily on radiotherapy days may be prescribed at the physician’s discretion.

#### Restaging

Four to 6 weeks after the last day of radiotherapy, pelvic MRI will be performed for local restaging and high-dose thoracoabdominal CT for restaging of possible distant metastases. Restaging imaging will be discussed during a dedicated MDT meeting in one of the expert centres. In the event of distant metastases or unresectable local disease, best palliative treatment will be offered. Patients with resectable disease will undergo surgery.

#### Surgery

Surgery will be performed by experienced surgical oncologists within 10–14 weeks after completion of chemoradiotherapy. The type of surgery will depend on the location of the recurrence and involvement of adjacent structures, and will be left to the discretion of the surgeon. When deemed necessary and feasible by the surgeon and radiation oncologist, intraoperative radiotherapy may be administered by either intraoperative electron beam radiotherapy or high-dose-rate intraoperative brachytherapy^38^^,^^39^.

#### Follow-up

Patients will be followed up at 3, 6, 9, 12, 15, 18, 21, 24, 27, 30, 33, 36, 42, 48, 54, and 60 months after surgery. At each follow-up point, a blood sample will be taken to determine the level of carcinoembryonic antigen (CEA). If the CEA level increases compared with the previous CEA level or the level rises above 5.0 µg/l during follow-up, high-dose thoracoabdominal CT will be performed. At 6, 12, 18, 24, 30, 36, 48, and 60 months after surgery, high-dose thoracoabdominal CT will be performed regardless of the CEA level.

#### Questionnaires

All participants will be asked to provide separate informed consent to receive validated quality of life questionnaires (European Organisation for Research and Treatment of Cancer QLQ-C30 and QLQ-CR29; EuroQol EQ-5D-5L™ (EuroQoL Group, Rotterdam, the Netherlands)). Patients will receive questionnaires at inclusion, and 3 and 12 months after surgery either by mail or digitally, according to their own preference.

#### Translational research

All participants will be asked to provide separate informed consent for collection of blood samples and/or tumour tissue for future translational research. If patients give such consent, an additional 20 ml blood will be drawn during regular blood draws before the start of induction chemotherapy (experimental arm only), before chemoradiotherapy, before surgery, 3 months after surgery, and once a year during 3 years of follow-up, resulting in seven samples per patient in the experimental arm and six per patient in the control arm. Tumour tissue will be collected by the pathologist, fresh frozen and stored until further use.

#### Central multidisciplinary team meetings

During the study inclusion period, a monthly central MDT meeting will be organized for quality control. All newly included patients will be discussed during this meeting, which has been designed as a teleconference. In addition, eligible patients will be discussed in the event of uncertainty about whether they meet the inclusion and/or exclusion criteria. Patients who are under treatment at the time of the central MDT meeting, or who have completed treatment, will be discussed only if there are remarkable findings, such as progression of disease resulting in unresectability.

### Outcomes

The primary outcome of the study is the proportion of patients with a clear resection margin. A resection margin is considered clear (R0), if there are no tumour cells in any of the resection surfaces as determined by microscopy (resection margin more than 0 mm).

Secondary outcomes are:

- 3- and 5-year local re-recurrence-free survival, defined as the interval between surgery and local re-recurrence;
- 3- and 5-year progression-free survival, defined as the interval between randomization and progression of local recurrence, local re-recurrence, distant metastases or death;
- 3- and 5-year metastasis-free survival, defined as the interval between randomization and development of distant metastases;
- 3- and 5-year disease-free survival, defined as the interval between surgery and local re-recurrence, distant metastases or death;
- 3- and 5-year overall survival, defined as the interval between randomization and death;
- pathological response, graded according to the Mandard grading system^40^;
- radiological response, scored according to the magnetic resonance tumour regression grade (mrTRG);
- compliance rate with induction chemotherapy (i.e., the number of patients receiving CAPOX, FOLFOX or FOLFIRI as initial regimen will be tabulated, and dose modification and reason will be summarized for each regimen);
- toxicity of induction chemotherapy, scored from day 1 of the first cycle of induction chemotherapy until 1 month after the final administration, and graded according to the Common Terminology Criteria for Adverse Events (CTCAE) version 5.0;
- compliance rate with chemoradiotherapy, calculated as the total radiotherapy dose received divided by the total planned dose;
- toxicity of chemoradiotherapy, scored from start of radiotherapy until 3 months after the final dose of radiotherapy, and graded according to CTCAE version 5.0;
- number of patients undergoing surgery;
- surgical characteristics (e.g., type of resection, ostomy, use of intraoperative radiotherapy, blood loss, duration of operation, intraoperative complications);
- major surgical morbidity rate scored from the date of surgery to 3 months after surgery, and graded according to the Clavien–Dindo classification^41^;
- quality of life, assessed with EQ-5D-5L™, QLQ-C30 and QLQ-CR29 questionnaires at inclusion, and at 3 and 12 months after surgery; and
- cost-effectiveness and cost–utility, based on Dutch costing guidelines for healthcare, the case report forms, and the EQ-5D-5L™ questionnaire.

### Sample size

Currently, an R0 resection is achieved in approximately 60 per cent of patients undergoing surgery after treatment with neoadjuvant chemoradiotherapy^16^^,^^17^. However, 25 per cent of patients who start neoadjuvant chemotherapy are not eligible for surgery owing to progressive disease: local progression, distant progression, or death from progression^12^. This means that an R0 resection is obtained in only 45 per cent of patients (75 per cent of 60 per cent) who start with intentionally curative treatment. The study hypothesis is that there will be a 15 per cent increase in the R0 resection rate (from 45 to 60 per cent) for patients in the experimental arm. A χ^2^ test with a 5 per cent two-sided significance level indicated that the study would have 80 per cent power to detect a significant difference of 15 per cent between the two groups (given that the percentage in the control group is 45 per cent) when the sample size in each group is 173 patients. With an expected dropout of 5 per cent, the total requirement was calculated as 364 patients.

### Statistical methods

Demographics, patient, and tumour characteristics will be presented for each treatment arm. Continuous data will be reported as mean (standard deviation) or median (interquartile range or 95 per cent confidence interval), depending on the distribution. Categorical data will be reported as count with percentage. All statistical tests will be two-sided and *P* < 0.050 will be classified as statistically significant. Patients initially randomized but considered ineligible afterwards, based on information that should have been available before randomization, will be excluded from all analyses.

Analysis of the primary endpoint of this study, the proportion of patients with an R0 resection, will be based on the intention-to-treat principle using Fisher’s exact test. In addition, a per-protocol analysis will be performed as a sensitivity analysis.

All survival curves will be constructed according to the Kaplan–Meier method, and the log rank test will be used to compare treatment arms, adjusting for stratification factors at randomisation (previous radiotherapy, previous systemic therapy, and expert centre). In addition, hazard ratios will be calculated using a Cox proportional hazards regression model, adjusting for stratification factors. Metastasis-free survival, progression-free survival, and overall survival will be based on the intention-to-treat group. Local re-recurrence free survival and disease-free survival analyses will include only patients who underwent surgery.

Data on surgical characteristics, histopathological characteristics, and major surgical morbidity will be presented by treatment arm, and will be derived only for patients who underwent surgery. The number of patients undergoing surgery will be analysed in the intention-to-treat population. Comparison between treatment arms will be done by means of Fisher’s exact test.

The absolute and relative incidence of toxicities related to the administration of induction chemotherapy or chemoradiotherapy will be presented by treatment arm, and analysed in all patients who received at least one dose of neoadjuvant chemotherapy (experimental arm) or chemoradiotherapy (control arm). Comparison between treatment arms will be done by means of Fisher’s exact test.

Comparison of health-related quality of life between the two treatment arms at baseline and over time will be performed by means of a random-effects regression model and will be based on the intention-to-treat group.

Incremental cost-effectiveness and cost–utility ratios will be calculated for the extra costs per additional surviving patient and the extra costs per additional quality-adjusted life year respectively. Non-parametric bootstrapping, drawing samples of the same size as the original samples and with replacement, will be applied to generate 95 per cent confidence intervals for (differences in) costs and health outcomes. Cost-effectiveness planes will be displayed and cost-effectiveness acceptability curves drawn for willingness-to-pay values up to €100 000.

### Data collection and management

A central study database (Netherlands Comprehensive Cancer Institute (IKNL), Utrecht, the Netherlands) with an electronic case report form will be used to record all data required to address the primary and secondary objectives. Local data management will be undertaken by the IKNL or an in-hospital qualified local data management team. Questionnaires will be collected centrally by the coordinating investigators and recorded using an ISO 27001-certified information security system (Research Manager, Deventer, Netherlands).

### Data safety monitoring board

A central data safety monitoring board (DSMB), consisting of a medical oncologist, a surgical oncologist, and a statistician, has been assigned to monitor the safety of study participants, and to protect the validity and credibility of the study. Members of the DMSB are independent and have no competing interest. After 100 patients have undergone surgery, the DSMB will review the safety data. Inclusion will be continued during interim analysis. At the interim analysis, the number of patients who cannot complete the full course of chemoradiotherapy and the number of patients with major postoperative morbidity (Clavien–Dindo grade at least III) will be tabulated and discussed. Examining these safety and logistical aspects will not affect the total sample size or the actual α level at final analysis. After the interim analysis, the DSMB will recommend to the trial steering committee (TSC) whether the study should be continued or terminated. Should the TSC decide not to fully implement the advice of the DSMB, it must explain to the medical ethical committee why (part of) the advice of the DSMB will not be followed.

### Harms

All serious adverse events (SAEs) or suspected unexpected serious adverse events (SUSARs) will be reported by the physician to the study coordinator within 24 h and without undue delay after obtaining knowledge of the event. The coordinating investigator will report SAEs through the web portal ToetsingOnline (https://www.toetsingonline.nl) to the medical ethical committee that approved the protocol. The time window for recording SAEs and SUSARs is from randomization until 3 months after surgery, or 1 month after the last day of neoadjuvant treatment for patients with progressive disease who did not undergo surgery. SAEs and SUSARs will be followed up until resolved or until a stable situation has been reached.

### Auditing

The study will be monitored by independent qualified monitors. The monitoring plan is based on the assessment that the study carries a moderate risk for the participants.

### Research ethics approval

This study was approved by the Medical Research Ethics Committees United, Nieuwegein, the Netherlands (R20.035), the Dutch Competent Authority (Centrale Commissie Mensgebonden Onderzoek, The Hague, the Netherlands; NL73593.100.20), and all institutional review boards of the participating study centres. The study will be submitted to the competent authorities, central ethical committees, and institutional review boards of the participating international centres.

### Protocol amendments

All substantial amendments will be notified to the (principal) investigators, institutional review boards of all study centres, the medical ethical committee, the competent authority, and trial registries.

### Consent and assent

Informed consent will be obtained by the treating physician in one of the expert centres. Patients will be allowed to provide separate permission for the collection of blood and/or tissue samples for translational research, and for receiving quality of life questionnaires.

### Confidentiality

Individual patient information obtained as a result of this study is considered confidential and its handling will conform with the Dutch Personal Data Protection Act (AVG). Patients’ confidentiality will be ensured by use of study numbers.

### Declaration of interests

The investigators declare no financial or other competing interests.

### Access to data

Access to the final data set is reserved for the central data manager, study statistician, coordinating investigator, and trial steering committee. There are no contractual agreements that limit this access.

### Ancillary and post-study care

The study has no provision for ancillary or post-study care.

### Dissemination policy

The results of this study will be dispersed by publishing the results in international peer-reviewed journals and by offering an abstract to international (surgical) oncological congresses. Any publication, abstract or presentation based on patients included in this study must be approved by the trial steering committee and coordinating investigator. The principal manuscript resulting from this study will be published by group authorship (PelvEx Collaborative).

## Discussion

This randomised controlled trial will investigate the role of induction chemotherapy in patients with LRRC. The results of this study will demonstrate whether or not induction chemotherapy has additional value in the treatment of patients with non-metastasized resectable LRRC with regard to the R0 resection rate; this group of patients has had a poor prognosis so far.

The rationale for R0 resection as the primary outcome in this study was based on the fact that R0 resection is the most important prognostic factor for survival in patients undergoing surgery for LRRC. Ultimately, an increase in R0 resection rate should lead to an improvement in local re-recurrence-free and overall survival. Because of the relatively rarity of LRRC as a result of improvements in the treatment of primary rectal cancer, and the fact that approximately 50 per cent of patients with LRRC will not be eligible for inclusion in this study owing to distant metastases or unresectable local disease, survival parameters could not be used as the primary outcome, as power calculations showed that the sample size would be unfeasible^42^^,^^43^.

The rationale for the induction chemotherapy regimen chosen in this study is based on studies in (metastatic) colorectal cancer. In those first-line studies^44–47^, doublet therapy led to better response rates and improved survival compared with monotherapy. Results of triplet therapy among patients with metastatic colorectal cancer have been conflicting^48^^,^^49^. In addition, although higher response rates have been observed for triple therapy compared with doublet therapy in patients with right-sided metastatic colorectal cancer, this has not been observed in patients with left-sided disease^50^^,^^51^. Moreover, triplet therapy is associated with more toxicity, and in patients with LRRC, in particular, toxicity of treatment is considered a major limitation often precluding curative treatment^52^. Therefore, doublet therapy is the treatment regimen of choice. As doublet therapy with capecitabine and oxaliplatin (CAPOX), 5-fluorouracil, leucovorin, and oxaliplatin (FOLFOX), and 5-fluorouracil, leucovorin, and irinotecan (FOLFIRI) have similar efficacy, all are incorporated in the present study protocol^46^^,^^53^^,^^54^.

There are three other ongoing trials investigating the optimal treatment for patients with LRRC. The French GRECCAR15 study (Chemotherapy Followed by Pelvic Reirradiation Versus Chemotherapy Alone as Pre-operative Treatment for Locally Recurrent Rectal Cancer; NCT03879109) is randomizing between induction chemotherapy, chemoreirradiation, and surgery *versus* induction chemotherapy and surgery in previously irradiated patients. The primary outcome measure is the R0 resection rate. The Japanese JCOG1801 study (Surgery Plus Chemo Versus Chemoradiotherapy Followed by Surgery Plus Chemo for Locally Recurrent Rectal Cancer; NCT04288999) is randomizing between surgery followed by adjuvant chemotherapy *versus* neoadjuvant chemoradiotherapy, surgery, and adjuvant chemotherapy (CAPOX/FOLFOX) in radiotherapy-naive patients, with local recurrence-free survival as the primary outcome measure. The Chinese NARC study (Efficacy and Safety Study of Neoadjuvant in Treating Patients With Resectable Local Recurrent Rectal Cancer; NCT01271192) is randomizing between surgery followed by adjuvant chemotherapy *versus* neoadjuvant chemoradiotherapy, surgery, and adjuvant chemotherapy, with overall survival as the primary outcome measure. The results of these studies will be actively monitored to assess whether their results have any implications for the present study protocol.

## Collaborators

Members of the PelvEx Collaborative: E. L. K. Voogt, S. Nordkamp, A. G. J. Aalbers, T. Buffart, G. J. Creemers, C. A. M. Marijnen, C. Verhoef, K. Havenga, F. A. Holman, M. Kusters, A. W. K. S. Marinelli, J. Melenhorst, N. Abdul Aziz, N. Abecasis, M. Abraham-Nordling, T. Akiyoshi, W. Alberda, M. Albert, M. Andric, E. Angenete, A. Antoniou, R. Auer, K. K. Austin, O. Aziz, R. P. Baker, M. Bali, G. Baseckas, B. Bebington, M. Bedford, B. K. Bednarski, G. L. Beets, R. G. H. Beets-Tan, M. Berbée, J. Berg, P. L. Berg, J. Beynon, S. Biondo, J. G. Bloemen, K. Boyle, L. Bordeianou, A. B. Bremers, M. Brunner, P. Buchwald, A. Bui, A. Burgess, D. Burling, E. Burns, N. Campain, S. Carvalhal, L. Castro, A. Caycedo-Marulanda, H. M. Ceha, K. K. L. Chan, G. J. Chang, M. Chang, M. H. Chew, A. K. Chok, P. Chong, H. K. Christensen, H. Clouston, M. Codd, D. Collins, A. J. Colquhoun, A. Corr, M. Coscia, M. Cosimelli, P. E. Coyne, A. S. L. P. Crobach, R. M. P. H. Crolla, R. S. Croner, L. Damjanovic, I. R. Daniels, M. Davies, R. J. Davies, C. P. Delaney, M. A. J. de Roos, J. H. W. de Wilt, M. D. den Hartogh, Q. Denost, P. Deseyne, C. Deutsch, R. de Vos tot Nederveen Cappel, M. de Vries, M. Dieters, D. Dietz, S. Domingo, M. Doukas, E. J. Dozois, M. Duff, T. Eglinton, J. M. Enrique-Navascues, E. Espin-Basany, M. D. Evans, B. Eyjólfsdóttir, M. Fahy, N. S. Fearnhead, S. Feshtali, K. Flatmark, F. Fleming, J. Folkesson, F. A. Frizelle, J. E. Frödin, M. A. Gallego, E. Garcia-Granero, J. L. Garcia-Sabrido, K. Geboes, L. Gentilini, M. L. George, V. George, L. Ghouti, F. Giner, N. Ginther, T. Glyn, R. Glynn, T. Golda, H. I. Grabsch, B. Griffiths, D. A. Harris, J. A.W. Hagemans, V. Hanchanale, D. P. Harji, R. M. Helewa, H. Helgason, G. Hellawell, A. G. Heriot, S. Heyman, D. Hochman, C. Hoff, W. Hohenberger, T. Holm, R. Hompes, K. Horsthuis, G. Hospers, J. Houwers, H. Iversen, J. T. Jenkins, S. Kaffenberger, G. V. Kandaswamy, S. Kapur, Y. Kanemitsu, G. Kats-Ugurlu, S. R. Kelley, D. S. Keller, M. E. Kelly, K. Keymeulen, M. S. Khan, H. Kim, H. J. Kim, C. E. Koh, N. F. M. Kok, R. Kokelaar, C. Kontovounisios, H. Ø. Kristensen, H. M. Kroon, S. Kumar, V. Lago, Z. Lakkis, T. Lamberg, S. G. Larsen, D. W. Larson, W. L. Law, S. Laurberg, P. J. Lee, M. M. Leseman-Hoogenboom, M. Limbert, M. L. Lydrup, A. Lyons, A. C. Lynch, C. Mantyh, K. L. Mathis, C. F. S. Margues, A. Martling, O. W. M. Meijer, W. J. H. J. Meijerink, A. Merchea, S. Merkel, A. M. Mehta, D. R. McArthur, F. D. McDermott, J. S. McGrath, S. Malde, A. Mirnezami, J. R.T. Monson, J. R. Morton, J. Nederend, I. Negoi, J. W. M. Neto, J. L. Ng, B. Nguyen, M. B. Nielsen, G. A. P. Nieuwenhuijzen, P. J. Nilsson, M. L. Nilsson, S. Oei, A. Oliver, S. T. O’Dwyer, V. Oppedijk, G. Palmer, E. Pappou, J. Park, D. Patsouras, G. Pellino, A. C. Peterson, H. M. U. Peulen, G. Poggioli, D. Proud, M. Quinn, A. Quyn, N. Rajendran, R. W. Radwan, S. Rasheed, P. C. Rasmussen, E. Rausa, S. E. Regenbogen, A. Renehan, M. C. Richir, R. Rocha, M. Rochester, J. Rohila, J. Rothbarth, M. Rottoli, C. Roxburgh, T. Rozema, B. Safar, P. M. Sagar, A. Sahai, A. Saklani, T. Sammour, R. Sayyed, A. M. P. Schizas, E. Schwarzkopf, V. Scripcariu, C. Selvasekar, I. Shaikh, D. Shida, A. Simpson, T. Skeie-Jensen, J. J. G. Slangen, N. J. Smart, P. Smart, J. J. Smith, P. Snaebjornsson, A. M. Solbakken, M. J. Solomon, M. M. Sørensen, L. Sorrentino, F. M. Speetjens, E. J. Spillenaar Bilgen, S. R. Steele, D. Steffens, K. Stitzenberg, L. Stocchi, N. A. Stylianides, T. Swartling, H. Sumrien, P. A. Sutton, T. Swartking, E. J. Tan, C. Taylor, P. P. Tekkis, J. Teras, V. Terpstra, R. Thurairaja, E. L. Toh, P. Tsarkov, Y. Tsukada, S. Tsukamoto, J. J. Tuech, W. H. Turner, J. B. Tuynman, E. B. van Duyn, W. M. U. van Grevenstein, N. C. T. van Grieken, L. Valkenburg-van Iersel, G. van Lijnschoten, E. van Meerten, G. H. van Ramshorst, H. L. van Westreenen, D. van Zoggel, W. Vasquez-Jimenez, L. A. Velema, E. Verdaasdonk, H. M. W. Verheul, K. S. Versteeg, G. Vizzielli, K. Uehara, C. Wakeman, S. Warrier, H. H. Wasmuth, K. Weber, M. R. Weiser, J. M. D. Wheeler, N. A. T. Wijffels, J. Wild, J. M. W E. Willems, M. Wilson, D. C. Winter, A. Wolthuis, M. L. Wumkes, H. Yano, B. Yip, J. Yip, R. N. Yoo, M. A. Zappa, D. D. E. Zimmerman, H. J. T. Rutten, J. W. A. Burger.

## Funding

This study is funded by the highly specialized care and research programme (TZO programme) of the Netherlands Organization for Health Research and Development (ZonMw).

*Disclosure.* The authors declare no conflict of interest.

## Notes

Members of the PelvEx Collaborative are co-authors of this study and are listed under the heading Collaborators.

## References

[1] Kapiteijn E , Marijnen CAM, Nagtegaal ID, Putter H, Steup WH, Wiggers T et al Preoperative radiotherapy combined with total mesorectal excision for resectable rectal cancer. N Engl J Med 2001;345:638–646 11547717 10.1056/NEJMoa010580

[2] Van Der Meij W , Rombouts AJM, Rutten H, Bremers AJA, De Wilt JHW. Treatment of locally recurrent rectal carcinoma in previously (chemo)irradiated patients: a review. Dis Colon Rectum 2016;59:148–156 26734974 10.1097/DCR.0000000000000547

[3] Bosman SJ , Holman FA, Nieuwenhuijzen GAP, Martijn H, Creemers GJ, Rutten HJT. Feasibility of reirradiation in the treatment of locally recurrent rectal cancer. Br J Surg 2014;101:1280–1289 25049111 10.1002/bjs.9569

[4] Valentini V , Morganti AG, Gambacorta MA, Mohiuddin M, Doglietto GB, Coco C et al Preoperative hyperfractionated chemoradiation for locally recurrent rectal cancer in patients previously irradiated to the pelvis: a multicentric phase II study. Int J Radiat Oncol Biol Phys 2006;64:1129–1139 16414206 10.1016/j.ijrobp.2005.09.017

[5] Dresen RC , Gosens MJ, Martijn H, Nieuwenhuijzen GA, Creemers GJ, Daniels-Gooszen AW et al Radical resection after IORT-containing multimodality treatment is the most important determinant for outcome in patients treated for locally recurrent rectal cancer. Ann Surg Oncol 2008;15:1937–1947 18389321 10.1245/s10434-008-9896-z PMC2467498

[6] Alberda WJ , Verhoef C, Schipper MEI, Nuyttens JJ, Rothbarth J, de Wilt JHW et al The importance of a minimal tumor-free resection margin in locally recurrent rectal cancer. Dis Colon Rectum 2015;58:677–685 26200682 10.1097/DCR.0000000000000388

[7] Westberg K , Palmer G, Hjern F, Nordenvall C, Johansson H, Holm T et al Population-based study of factors predicting treatment intention in patients with locally recurrent rectal cancer. Br J Surg 2017;104:1866–1873 29023631 10.1002/bjs.10645

[8] Westberg K , Palmer G, Hjern F, Johansson H, Holm T, Martling A. Management and prognosis of locally recurrent rectal cancer—a national population-based study. Eur J Surg Oncol 2018;44:100–107 29224985 10.1016/j.ejso.2017.11.013

[9] Kusters M , Dresen RC, Martijn H, Nieuwenhuijzen GA, van de Velde CJH, van den Berg HA et al Radicality of resection and survival after multimodality treatment is influenced by subsite of locally recurrent rectal cancer. Int J Radiat Oncol Biol Phys 2009;75:1444–1449 19395199 10.1016/j.ijrobp.2009.01.015

[10] Holman FA , Bosman SJ, Haddock MG, Gunderson LL, Kusters M, Nieuwenhuijzen GAP et al Results of a pooled analysis of IOERT containing multimodality treatment for locally recurrent rectal cancer: results of 565 patients of two major treatment centres. Eur J Surg Oncol 2017;43:107–117 27659000 10.1016/j.ejso.2016.08.015

[11] Mannaerts G , Martijn H, Rutten H, Hanssens P, Wiggers T. Local tumor control and (disease-free) survival after surgery with pre- and intraoperative radiotherapy for primary non-resectable rectal carcinoma and local recurrence. Ned Tijdschr Geneeskd 2001;145:1460–1466 11503316

[12] Hagemans JAW , van Rees JM, Alberda WJ, Rothbarth J, Nuyttens JJME, van Meerten E et al Locally recurrent rectal cancer; long-term outcome of curative surgical and palliative treatment of 447 consecutive patients in a tertiary referral centre. Eur J Surg Oncol 2020;46:448–454 31761506 10.1016/j.ejso.2019.10.037

[13] Camilleri-Brennan J , Steel R. The impact of recurrent rectal cancer on quality of life. Eur J Surg Oncol 2001;27:349–353 11417978 10.1053/ejso.2001.1115

[14] Glynne-Jones R , Wyrwicz L, Tiret E, Brown G, Rödel C, Cervantes A et al Rectal cancer: ESMO Clinical Practice Guidelines for diagnosis, treatment and follow-up. Ann Oncol 2018;29:263 10.1093/annonc/mdy161 29741565

[15] Guren MG , Undseth C, Rekstad BL, Brændengen M, Dueland S, Spindler KLG et al Reirradiation of locally recurrent rectal cancer: a systematic review. Radiother Oncol 2014;113:151–157 25613395 10.1016/j.radonc.2014.11.021

[16] Nielsen M , Rasmussen P, Pedersen B, Hagemann-Madsen R, Lindegaard J, Laurberg S. Early and late outcomes of surgery for locally recurrent rectal cancer: a prospective 10-year study in the total mesorectal excision era. Ann Surg Oncol 2015;22:2677–2684 25564165 10.1245/s10434-014-4317-y

[17] Yu SKT , Bhangu A, Tait DM, Tekkis P, Wotherspoon A, Brown G. Chemoradiotherapy response in recurrent rectal cancer. Cancer Med 2014;3:111–117 24403010 10.1002/cam4.169 PMC3930395

[18] Milas L , Hunter NR, Mason KA, Milross CG, Saito Y, Peters LJ. Role of reoxygenation in induction of enhancement of tumor radioresponse by paclitaxel. Cancer Res 1995;55:3564–3568 7627965

[19] Voogt ELK , van Zoggel DMGI, Kusters M, Nieuwenhuijzen GAP, Bloemen JG, Peulen HMU et al Improved outcomes for responders after treatment with induction chemotherapy and chemo(re)irradiation for locally recurrent rectal cancer. Ann Surg Oncol 2020;27:3503–3513 32193717 10.1245/s10434-020-08362-4

[20] Hospers G , Bahadoer RR, Dijkstra EA, van Etten B, Marijnen C, Putter H et al Short-course radiotherapy followed by chemotherapy before TME in locally advanced rectal cancer: the randomized RAPIDO trial. J Clin Oncol 2020;38:4006

[21] Braun MS , Seymour MT. Balancing the efficacy and toxicity of chemotherapy in colorectal cancer. Ther Adv Med Oncol 2011;3:43–52 21789155 10.1177/1758834010388342 PMC3126034

[22] Hardiman KM , Antunez AG, Kanters A, Schuman AD, Regenbogen SE. Clinical and pathological outcomes of induction chemotherapy before neoadjuvant radiotherapy in locally‐advanced rectal cancer. J Surg Oncol 2019;120:308–315 30993710 10.1002/jso.25474 PMC6635055

[23] Chau I , Brown G, Cunningham D, Tait D, Wotherspoon A, Norman AR et al Neoadjuvant capecitabine and oxaliplatin followed by synchronous chemoradiation and total mesorectal excision in magnetic resonance imaging-defined poor-risk rectal cancer. J Clin Oncol 2006;24:668–674 16446339 10.1200/JCO.2005.04.4875

[24] Cercek A , Goodman KA, Hajj C, Weisberger E, Segal NH, Reidy-Lagunes DL et al Neoadjuvant chemotherapy first, followed by chemoradiation and then surgery, in the management of locally advanced rectal cancer. J Natl Compr Canc Netw 2014;12:513–519 24717570 10.6004/jnccn.2014.0056 PMC5612781

[25] Masi G , Vivaldi C, Fornaro L, Lonardi S, Buccianti P, Sainato A et al Total neoadjuvant approach with FOLFOXIRI plus bevacizumab followed by chemoradiotherapy plus bevacizumab in locally advanced rectal cancer: the TRUST trial. Eur J Cancer 2019;110:32–41 30739838 10.1016/j.ejca.2019.01.006

[26] Schou JV , Larsen FO, Rasch L, Linnemann D, Langhoff J, Høgdall E et al Induction chemotherapy with capecitabine and oxaliplatin followed by chemoradiotherapy before total mesorectal excision in patients with locally advanced rectal cancer. Ann Oncol 2012;23:2627–2633 22473488 10.1093/annonc/mds056

[27] Fernández-Martos C , Pericay C, Aparicio J, Salud A, Safont M, Massuti B et al Phase II, randomized study of concomitant chemoradiotherapy followed by surgery and adjuvant capecitabine plus oxaliplatin (CAPOX) compared with induction CAPOX followed by concomitant chemoradiotherapy and surgery in magnetic resonance imaging-defined, l. J Clin Oncol 2010;28:859–865 20065174 10.1200/JCO.2009.25.8541

[28] Fernandez-Martos C , Garcia-Albeniz X, Pericay C, Maurel J, Aparicio J, Montagut C et al Chemoradiation, surgery and adjuvant chemotherapy *versus* induction chemotherapy followed by chemoradiation and surgery: long-term results of the Spanish GCR-3 phase II randomized trial. Ann Oncol 2015;26:1722–1728 25957330 10.1093/annonc/mdv223

[29] Garcia-Aguilar J , Chow OS, Smith DD, Marcet JE, Cataldo PA, Varma MG et al; Timing of Rectal Cancer Response to Chemoradiation Consortium. Effect of adding mFOLFOX6 after neoadjuvant chemoradiation in locally advanced rectal cancer: a multicentre, phase 2 trial. Lancet Oncol 2015;16:957–966 26187751 10.1016/S1470-2045(15)00004-2 PMC4670237

[30] Tang J , Wu X, Bai Y, Gao Y, Jiang W, Kong L et al Long-Term outcome of oxaliplatin and capecitabine (XELOX) concomitant with neoadjuvant radiotherapy and extended to the resting period in high risk locally advanced rectal cancer. J Cancer 2018;9:1365–1370 29721045 10.7150/jca.23874 PMC5929080

[31] Yu X , Wang QX, Xiao WW, Chang H, Zeng ZF, Lu ZH et al Neoadjuvant oxaliplatin and capecitabine combined with bevacizumab plus radiotherapy for locally advanced rectal cancer: results of a single-institute phase II study. Cancer Commun (Lond) 2018;38:24 29784042 10.1186/s40880-018-0294-z PMC5993137

[32] Golo D , But-Hadzic J, Anderluh F, Brecelj E, Edhemovic I, Jeromen A et al Induction chemotherapy, chemoradiotherapy and consolidation chemotherapy in preoperative treatment of rectal cancer—long-term results of phase II OIGIT-01 trial. Radiol Oncol 2018;52:267–274 30210040 10.2478/raon-2018-0028 PMC6137354

[33] Maréchal R , Vos B, Polus M, Delaunoit T, Peeters M, Demetter P et al Short course chemotherapy followed by concomitant chemoradiotherapy and surgery in locally advanced rectal cancer: a randomized multicentric phase II study. Ann Oncol 2012;23:1525–1530 22039087 10.1093/annonc/mdr473

[34] Cercek A , Roxburgh CSD, Strombom P, Smith JJ, Temple LKF, Nash GM et al Adoption of total neoadjuvant therapy for locally advanced rectal cancer. JAMA Oncol 2018;4:e180071 29566109 10.1001/jamaoncol.2018.0071 PMC5885165

[35] Calvo FA , Sole CV, Serrano J, Del Valle E, Rodriguez M, Muñoz-Calero A et al Preoperative chemoradiation with or without induction oxaliplatin plus 5-fluorouracil in locally advanced rectal cancer: long-term outcome analysis. Strahlenther Onkol 2014;190:149–157 24306062 10.1007/s00066-013-0469-0

[36] Kusters M , Bosman SJ, Van Zoggel DMGI, Nieuwenhuijzen GAP, Creemers GJ, Van den Berg HA et al Local recurrence in the lateral lymph node compartment: improved outcomes with induction chemotherapy combined with multimodality treatment. Ann Surg Oncol 2016;23:1883–1889 26842492 10.1245/s10434-016-5098-2

[37] van Zoggel DMGI , Bosman SJ, Kusters M, Nieuwenhuijzen GAP, Cnossen JS, Creemers GJ et al Preliminary results of a cohort study of induction chemotherapy-based treatment for locally recurrent rectal cancer. Br J Surg 2018;105:447–452 29168556 10.1002/bjs.10694

[38] Biggs P , Willet CG, Rutten HJT, Ciocca M, Gunderson LL, Calvo FA. Intraoperative electron beam irradiation: physics and techniques. In: Gunderson LL, Willett CG, Calvo FA, Harrison LB (eds). Intraoperative Irradiation: Techniques and Results (2nd edn). Totowa: Humana Press, 2011, 51–72

[39] Furhang EE , Silanpaa JK, Hu KS, Harrison LB. HDR-IORT: physics and techniques. In: Gunderson LL, Willett CG, Calvo FA, Harrison LB (eds). Intraoperative Irradiation: Techniques and Results (2nd edn). Totowa: Humana Press, 2011, 73–84

[40] Mandard AM , Dalibard F, Mandard JC et al Pathologic assessment of tumor regression after preoperative chemoradiotherapy of esophageal carcinoma. Clinicopathologic correlations. Cancer 1994;73:2680–2686 8194005 10.1002/1097-0142(19940601)73:11<2680::aid-cncr2820731105>3.0.co;2-c

[41] Clavien PA , Barkun J, de Oliveira ML, Vauthey JN, Dindo D, Schulick RD et al The Clavien–Dindo classification of surgical complications five-year experience. Ann Surg 2009;250:187–196 doi:10.1097/SLA.0b013e3181b13ca2 19638912 10.1097/SLA.0b013e3181b13ca2

[42] Heriot AG , Tekkis PP, Darzi A, Mackay J. Surgery for local recurrence of rectal cancer. Colorectal Dis 2006;8:733–747 17032318 10.1111/j.1463-1318.2006.01018.x

[43] van den Brink M , Stiggelbout AM, van den Hout WB, Kievit J, Klein Kranenbarg E, Marijnen CAM et al Clinical nature and prognosis of locally recurrent rectal cancer after total mesorectal excision with or without preoperative radiotherapy. J Clin Oncol 2004;22:3958–3964 15459218 10.1200/JCO.2004.01.023

[44] Douillard JY , Cunningham D, Roth AD, Navarro M, James RD, Karasek P et al Irinotecan combined with fluorouracil compared with fluorouracil alone as first-line treatment for metastatic colorectal cancer: a multicentre randomised trial. Lancet 2000;355:1041–1047 10744089 10.1016/s0140-6736(00)02034-1

[45] de Gramont A , Figer A, Seymour M, Homerin M, Hmissi A, Cassidy J et al Leucovorin and fluorouracil with or without oxaliplatin as first-line treatment in advanced colorectal cancer. J Clin Oncol 2000;18:2938–2947 10944126 10.1200/JCO.2000.18.16.2938

[46] Tournigand C , André T, Achille E, Lledo G, Flesh M, Mery-Mignard D et al FOLFIRI followed by FOLFOX6 or the reverse sequence in advanced colorectal cancer: a randomized GERCOR study. J Clin Oncol 2004;22:229–237 14657227 10.1200/JCO.2004.05.113

[47] Grothey A , Sargent D, Goldberg RM, Schmoll HJ. Survival of patients with advanced colorectal cancer improves with the availability of fluorouracil–leucovorin, irinotecan, and oxaliplatin in the course of treatment. J Clin Oncol 2004;22:1209–1214 15051767 10.1200/JCO.2004.11.037

[48] Falcone A , Ricci S, Brunetti I, Pfanner E, Allegrini G, Barbara C et al Phase III trial of infusional fluorouracil, leucovorin, oxaliplatin, and irinotecan (FOLFOXIRI) compared with infusional fluorouracil, leucovorin, and irinotecan (FOLFIRI) as first-line treatment for metastatic colorectal cancer: the gruppo oncologico nor. J Clin Oncol 2007;25:1670–1676 17470860 10.1200/JCO.2006.09.0928

[49] Souglakos J , Androulakis N, Syrigos K, Polyzos A, Ziras N, Athanasiadis A et al FOLFOXIRI (folinic acid, 5-fluorouracil, oxaliplatin and irinotecan) *vs* FOLFIRI (folinic acid, 5-fluorouracil and irinotecan) as first-line treatment in metastatic colorectal cancer (MCC): a multicentre randomised phase III trial from the Hellenic Oncology Research Grooup (HORG). Br J Cancer 2006;94:798–805 16508637 10.1038/sj.bjc.6603011 PMC2361370

[50] Cremolini C , Loupakis F, Antoniotti C, Lupi C, Sensi E, Lonardi S et al FOLFOXIRI plus bevacizumab *versus* FOLFIRI plus bevacizumab as first-line treatment of patients with metastatic colorectal cancer: updated overall survival and molecular subgroup analyses of the open-label, phase 3 TRIBE study. Lancet Oncol 2015;16:1306–1315 26338525 10.1016/S1470-2045(15)00122-9

[51] Cremolini C , Antoniotti C, Lonardi S, Bergamo F, Cortesi E, Tomasello G et al Primary tumor sidedness and benefit from FOLFOXIRI plus bevacizumab as initial therapy for metastatic colorectal cancer. Retrospective analysis of the TRIBE trial by GONO. Ann Oncol 2018;29:1528–1534 29873679 10.1093/annonc/mdy140

[52] Falcone A , Cremolini C, Masi G, Lonardi S, Zagonel V, Salvatore L et al FOLFOXIRI/bevacizumab *versus* FOLFIRI/bevacizumab as first-line treatment in unresectable metastatic colorectal cancer (mCRC) patients: reults of the phase III TRIBE trial by GONO group. J Clin Oncol 2013;31:3505

[53] Arkenau HT , Arnold D, Cassidy J, Diaz-Rubio E, Douillard JY, Hochster H et al Efficacy of oxaliplatin plus capecitabine or infusional fluorouracil/leucovorin in patients with metastatic colorectal cancer: a pooled analysis of randomized trials. J Clin Oncol 2008;26:5910–5917 doi:10.1200/JCO.2008.16.7759 19018087 10.1200/JCO.2008.16.7759

[54] Sobrero A , Bennicelli E. Chemotherapy: which drug and when? Ann Oncol 2010;21:vii130–vii133 20943605 10.1093/annonc/mdq293

